# Periscope

**Published:** 1882-02

**Authors:** 


### PERISCOPE.

American Observer, November, 1881:—Opens with pernicious advice as to the use of *ergot* and the *per-sulphate of iron* in *post-partum* hemorrhage. No mention is made of the many homœopathic remedies which have accomplished such wonders in this dangerous disease, probably because the writer knew not of them! We mention this subject as such advice is just now common among a certain class of homœopaths. In an article on "Diphtheria," the following is given: "Dr. C. Lippe gives the specific indications for *Lac. caninum* as follow: The ulcers go from one side to the other and *back again;* the ulceration has a glistening, shining appearance (*Apis*); the swelling of the gland changes sides and is painful to touch, and the nasal discharge excoriates the nostrils and upper lip (*Arum triph.*)"

The following *clinical observations* in cases cured by *Lac. can.* are by Dr. C. F. Nichols, of Boston:

"Pains in limbs, small of back and head disappear, and the throat becomes more painful but looks better. Often the ulcers increase in size or number, but the neighboring membrane looks clearer; worse by empty deglutition; throat feels stiff; relief after drinking, warm or cold, no thirst but dry mouth; pain pushes toward left ear; right tonsil raw, swollen, gray-white membrane there and on the fauces; epistaxis when speaking or swollowing, in one case; sweat all over; great exhaustion with "poisoned feeling;" frequent micturition, urine dark; restless, legs and whole body; face burns, dry; constant spitting, drooping—in one case, a man, very quickly relieved; imagines he wears somebody else's nose—same case; ulcers small, round or irregular, gray-white; voice hoarse; interrupted by weakness and hoarseness. Several cases cured resembled *Lachesis.*"

American Homœopath, November:—A paper read before the Pennsylvania Homœopathic Society by Dr. E. A. Farrington, on "*Bryonia and Rhus tox. Considered in Reference to the Effects of Motion,*" is quoted. Dr. F. says: "a general characteristic symptom of *Bryonia* is undoubtedly worse from motion. But such a fact ought not to prevent our employing the drug when an exactly opposite condition obtains, if other symptons aid our choice." The same may be said of the *Rhus tox.* desire for, and improvement from motion. But this peculiarity (that is a drug being indicated in cases where its prominent characteristic symptom is lacking) pertains to many, if not all drugs. Thus we have known *Pulsatilla* to cure in cases where heat (of room and clothing) was desired and improved the symptoms. So also has *Calcarea* cured menstrual disorders in cases where scanty menses where observed. In short, we should remember it is the totality of the characteristic symptoms that must guide us in selecting our remedy, not any *one* symptom. These drug peculiarities, if we may so term them, are most clearly indicated in Bœnninghausen's invaluable "Therapeutic Pocket Book."

Dr. Farrington adds: "In view of these facts, it concerns us not to select a drug merely because its prominent modalities are present in the case to be treated. He is a routinist who uses specifics; and he also is a routinist who prescribes for one symptom.

"Our journals teem with reports of so-called cures, in which the only apparent similarity between disease and drug is a single modality; such as, worse left side, *Lachesis*; wants to lie perfectly still, *Bryonia*.

"Rather let us follow the Master, who enjoins us to draw our characteristic picture from the totality of the symptoms." Sound and sensible advice.

Dr. C. H. Brace writes on "Intermittent Fever," from Cumberland, Md., a great "ague" country, yet he finds "that the law of similia similibus curantur applies to the treatment of malaria as well as to all the other ills that flesh is heir to." If patients have been dosed previously with *Quinine*, Dr. Brace finds *Ferrum* 6x an excellent antidote; he gives his indicated remedy *during* the chill and fever, rather than between paroxysms.

The following, taken from the *Hom. World*, and written by its accomplished editor, is interesting:

A Case of Cataract, much Improved by Medicine.—Mrs. ——, aet. 81. Thinking her case hopeless, principally on account of her advanced age, I did not enter with my wonted minuteness into her case, but gave *Chilidonium* 14, five drops in water night and morning, on pathological grounds.

Feb. 2d, 1881.—She came and said she felt more comfortable in her *mouth*, her tongue being less hard and stiff; vision the same (*i. e.*, much impaired, reading impossible; can barely recognize one in the street.) * * * I now went into her case with great care. I found that she had occasional diplopia, and things seemed further off than they really were.

But the thing that had long distressed her was this: *On awaking in the morning her tongue was as hard and stiff as a board.* That this should have any connection with the cataractous lenses was not apparent; still it was the *most constant peculiar*, and *characteristic symptom*, and moreover a very distressing one. R. *Sulphur iodatum* (see symptom 40, in Allen's Encyclopædia), six grains of the 4th cent. trituration, every night at bed time.

March 21.—Hardness and stiffness of the tongue *gone;* had had it two years. Sees decidedly better at a distance.

July.—Vision much improved; can now read an article in a newspaper. R. *Iodium.*

August.—Sees so well that she does not propose to continue the treatment any longer.

Allen's "Symptom Register" gives under hardness of tongue; *Atro.*, *Hyos.*, *Kali iod.*, *Merc.*, *Sul. iod.* Under Stiffness, of these only *Hyos.* is found. Stiffness in morning, *Amm. br.*, *Dios.*; on waking, *Coc. c.*, (3 A. M.), *Natr. m.*

MEDICAL COUNSELOR, Nov. 16th.—Dec. 21st: Translated from the *Allg. Hom. Zeitg.*, is a case of caries of wrist and elbow cured by *Silicea.*

A girl, aged 18, had the itch soon after her birth which was suppressed by local treatment. When 16 years old, she had caries of left foot, which was amputated. After three months, the caries attacked the elbow-joint. Nine months after she presented the following symptoms: The elbow and wrist of right arm are very much swollen, red and hot. A large number of fistulæ give exit to a fœtid, sanious pus, and run into the joints, where the bones are necrosed, conveying to the probe a decided roughness. She has pain day and night. Pains are stitching, drawing and tearing. She moans and sobs constantly from the severity of the pain, which is increased by any pressure, touch or motion. Great thirst; poor appetite; hectic fever, profuse sweats. Gave one dose *Silicea* 30. The pains disappeared entirely and never returned for three years. She took one dose of *Silicea* every forty days. The destroyed bone was reproduced; the joints, of course, remained immovable, but in course of time she got limited motion. She remained well.

We have cured several cases of inflammation, where beside the usual throbbing pains of *Silicea*, there was the annoying *itching* in the part.

Two letters from Hahnemann to his friend Stapf are translated by the editor. If we are not mistaken, we read these letters in the 3rd volume of the *British Journal of Homœopathy.* They are well worth reproducing.

HAHNEMANNIAN MONTHLY, DECEMBER.—Dr. Farrington, in his *Studies in Materia Medica*, writes on Spongia tosta, giving the following: Croup, with harsh, barking cough, worse the first part of night; sawing respiration; child arouses from sleep startled, suffocating, with long-drawn breaths and barking cough; better holding head back. Caused by exposure to dry, cold winds (*Hepar*). Its croup is spasmodic, and is characterized by little fever, here differing from *Aconite.*

*Aconite* is preferable when, in croup, the child arouses with suffocation, cough is harsh, barking; face expressive of anxious fear; skin hot or bathed in sweat. Caused by exposure to cold wind. If the anxiety or the heat continues or returns the next night, persist with the same remedy (*Aconite*), but if the respiration becomes more sawing or labored, as if forced through a sponge, the anxiety present but less marked, the fever somewhat diminished, sputum still absent or scant, change to *Spongia.*

*Hepar* often follows *Spongia* when the cough is accompanied with a mucous sound, though it preserves its barking tone. This frequently occurs after midnight, towards morning. Hence *Hepar* is generally required later than *Spongia.* It must be remembered, however, that *Hepar* also develops a tedious, dry, barking cough, coming on as soon as the child lies down at night. This cough, common in croupy children, *Hepar* relieves promptly. *Causticum* is a good substitute in some cases of catarrhal or spasmodic croup. The child, while inspiring, chokes as if clutched by the throat; raw, burning feeling, in a streak, down the course of the trachea. *Kali bromatum* is not to be forgotten in cases of weak, nervous children, who arouse with a dry, spasmodic cough, which greatly frightens them, causing them to cry out in terror. It has several times happened in our experience that *Drosera* was needed for a barking evening cough, simulating that of *Spongia.* The coughs were frequent and persistent, combining the spasmodic with the croupy sound. *Bromine* and *Iodine* may follow. The first suits in membranous croup, whether diphtheritic or not; the larynx seems to be full of loose mucus. The child is aroused suddenly choking; a drink of water relieves temporarily. *Iodine* causes a dry cough, with noisy respiration and fever. Child tears at throat (*Aconite,* grasps throat); raises large flakes of tough, but not stringy exudation.

"*Phosphorus* resembles *Spongia* in tuberculosis. Both are indicated in youth, with weakness and rush of blood to chest; but the cough and laryngeal symptoms differ. More nearly related are *Spongia* and *Hepar.* They suit in cases which cannot tolerate dry, cold air. The former is needed when cough is dry, hard; and worse before 12 P. M.; the latter when cough sounds hard, but there is phlegm in larynx and bronchi; in morning on going into the open air, his throat fills with mucus, making voice husky."

